# Chronic Exposure to Subtherapeutic Antibiotics Aggravates Ischemic Stroke Outcome in Mice

**DOI:** 10.1016/j.ebiom.2017.09.002

**Published:** 2017-09-06

**Authors:** Xiao-Hui Dong, Cheng Peng, Yu-Yi Zhang, Yu-long Tao, Xia Tao, Chuan Zhang, Alex F. Chen, He-Hui Xie

**Affiliations:** a School of Public Health, Hongqiao International Institute of Medicine, Shanghai Jiao Tong University School of Medicine, Shanghai 200025, China; b Department of Pharmacy, Shanghai East Hospital, Tongji University School of Medicine, Shanghai 200120, China; c Third Xiangya Hospital, The Institute of Vascular Disease and Translational Medicine, Central South University, Changsha, Hunan 410013, China; d Department of Pharmacy, Shanghai Changzheng Hospital, The Second Military Medical University, Shanghai 200120, China; e Department of Identification of Traditional Chinese Medicine, The Second Military Medical University, Shanghai 200433, China

**Keywords:** Antibiotics, Stroke, Endothelial progenitor cells, Angiogenesis

## Abstract

Subtherapeutic antibiotics have been widely used in agriculture since the 1950s, which can be accumulated in human body through various approaches and may have long-term consequences. However, there is limited information about the link between chronic subtherapeutic antibiotic exposure and the outcome of ischemic brain injury. Here we showed that long-term treatment with subtherapeutic chlortetracycline, penicillin or vancomycin, which were widely used in agriculture approved by US Food and Drug Administration (FDA), could impair EPC functions, reduce ischemic brain angiogenesis and aggravate cerebral ischemic injury and long-term stroke outcomes in mice. In addition, transplantated EPCs from chronic antibiotic-treated mice showed a lower therapeutic effect on cerebral ischemic injury reduction and local angiogenesis promotion compared to those from control mice, and EPCs from the donor animals could integrate into the recipient ischemic brain in mice. Furthermore, transplanted EPCs might exert paracrine effects on cerebral ischemic injury reduction in mice, which could be impaired by chronic antibiotic exposure. In conclusion, chronic subtherapeutic antibiotic exposure aggravated cerebral ischemic injury in mice, which might be partly attributed to the impairment of both EPC-mediated angiogenesis and EPCs' paracrine effects. These findings reveal a previously unrecognized impact of chronic subtherapeutic antibiotic exposure on ischemic injury.

## Highlights

• Chronic exposure to subtherapeutic antibiotics aggravates long-term stroke outcome in mice.
• Chronic exposure to subtherapeutic antibiotics impairs endothelial progenitor cell functions in mice.
• Chronic exposure to subtherapeutic antibiotics reduces ischemic brain angiogenesis in mice.

Subtherapeutic antibiotics have been widely used in agriculture since the 1950s, which can be accumulated in human body through various approaches and may have long-term consequences. However, there is limited information about the link between chronic subtherapeutic antibiotic exposure and the outcome of ischemic brain injury. Here we showed that chronic treatment with subtherapeutic chlortetracycline, penicillin or vancomycin, which were widely used in agriculture approved by US Food and Drug Administration (FDA), could impair endothelial progenitor cells-mediated angiogenesis and aggravate long-term stroke outcome in mice. These findings reveal a previously unrecognized impact of chronic subtherapeutic antibiotic exposure on ischemic injury.

## 1 Introduction

It has been demonstrated that the administration of low doses of antibacterial agents promotes the growth of farm animals, and the effects are observed with many different classes of antibacterial agents (including macrolides, tetracyclines and penicillins, etc), indicating that the activity is not an agent-specific side effect. Accordingly, the largest use of antibiotics and related antimicrobial substances is within farms in the United States, with low doses fed to large numbers of animals used for food production to increase weight gain by as much as 15% (Butaye et al., 2003; Cho et al., 2012). These antibiotics can be accumulated in human body through various approaches. There is increasing concern that antibiotic exposure may have long-term consequences (Blaser and Falkow, 2009; Cho et al., 2012; Manichanh et al., 2010). Recent research has found that early-life subtherapeutic antibiotic treatment increased fat mass, altered metabolic hormones, hepatic metabolism, and microbiota composition in mice (Cho et al., 2012). Early life is a critical period for metabolic development, microbiota disruption during this window could lead to changes in body composition (Cho et al., 2012; Cox et al., 2014). In humans, early-life microbiota disruption, either due to delivery by Caesarian section or antibiotics, is associated with increased risk of overweight status later in childhood (Cho et al., 2012; Cox et al., 2014).

The metabolic disorders (including obesity, diabetes, etc) are associated with the impairment of endothelial progenitor cells (EPCs) and EPC-mediated ischemic angiogenesis, and can increase the risk of cardiovascular and cerebrovascular diseases such as stroke (a devastating disease and the major cause of mortality and morbidity worldwide) (Dong et al., 2015; Jensen et al., 2014; Zhao et al., 2013). EPCs are precursor cells of vascular endothelial cells, which can be mobilized from the bone marrow to the injury site to promote endothelial regeneration and neovascularization, and have been used to successfully improve function recovery in ischemic organs including brain after ischemic injury. Recent reports showed that EPCs may serve as a new marker for stroke outcomes (Zhao et al., 2013; Fan et al., 2010; Marrotte et al., 2010). Moreover, the endothelium controls several vascular functions, including vascular tone and permeability, thrombosis, hemostasis, and angiogenesis, and the integrity and function of the endothelium plays an important role in the prevention of cerebrocardiovascular diseases (Zhao et al., 2013; Fan et al., 2010; Marrotte et al., 2010). Thus, EPC dysfunction and the subsequent abnormality of endothelial regeneration and EPC-mediated ischemic angiogenesis may influence the susceptibility to cerebral ischemic injury.

On the basis of these findings, this study sought to determine the effect of chronic exposure to subtherapeutic antibiotic dosages from early life on the EPC-mediated ischemic angiogenesis and cerebral ischemic injury in mice. Among antibiotics widely used in agriculture approved by US Food and Drug Administration (FDA) (Cho et al., 2012), chlortetracycline, penicillin and vancomycin were adopted as the representatives in the present study.

A recently published study in *Nature Medicine* showed that treatment with therapeutic doses of antibiotics from drinking water (1000 mg/l or 500 mg/l) for 2 weeks could reduce ischemic brain injury via inducing alterations in the intestinal flora in mice (Benakis et al., 2016). However, surprisingly, the present work demonstrated, for the first time, that chronic (6 months) exposure to subtherapeutic antibiotics from drinking water (6.67 mg/l, in the mid-range of FDA-approved levels for subtherapeutic antibiotic use in agriculture) significantly aggravated cerebral ischemic injury in mice, which might be partly attributed to the impairment of both EPC-mediated angiogenesis and EPCs' paracrine effects.

## 2 Materials and Methods

### 2.1 Animals and Antibiotics Treatment

Female C57BL/6J mice (Sino-British SIPPR/BK Lab Animal Ltd., Shanghai, China) were obtained at weaning (3 weeks, 10 to 13 g) and allowed to adjust to the animal facility for 1 week. Mice were weighed at the beginning of each experiment and distributed five per cage so that mean weights in each cage were equal. The animals were allowed ad libitum access to food and water and were maintained on a 12-h light/dark cycle and fed standard laboratory chow (No. M01-F, Shanghai Pu Lu Teng Biological Technology Co., Ltd., China). Beginning at 4 weeks of age, mice were given chlortetracycline (Chl, Sigma), penicillin VK (Phe, Sigma), vancomycin (Van, Sigma) or water (Control) via drinking water for 6 months. Doses were adapted from the US Food and Drug Administration (FDA) Green Book and generally were in the mid-range of those approved for use in agriculture (Cho et al., 2012; Cox et al., 2014). To simplify administration, the antibiotics were added to drinking water (6.67 mg/l) to deliver approximately 1 μg antibiotic per g body weight of the mice in the cage, based on the calculation that daily water intake is proportional to body weight (Cho et al., 2012; Cox et al., 2014). In previous studies, the daily water intake in C57BL/6J mice averaged 15 ml water per 100 g body weight (Cho et al., 2012; Cox et al., 2014; Harkness and Wagner, 1989). Water containers were changed twice weekly to supply fresh antibiotics.

The body weight was monitored every 4 weeks, and fasting blood glucose levels were measured before and after 6 months of treatment. All animals received humane care, and the experimental procedures were in compliance with the institutional animal care guidelines. All the experiments were performed in a random and blind fashion.

**Fig. 1:**
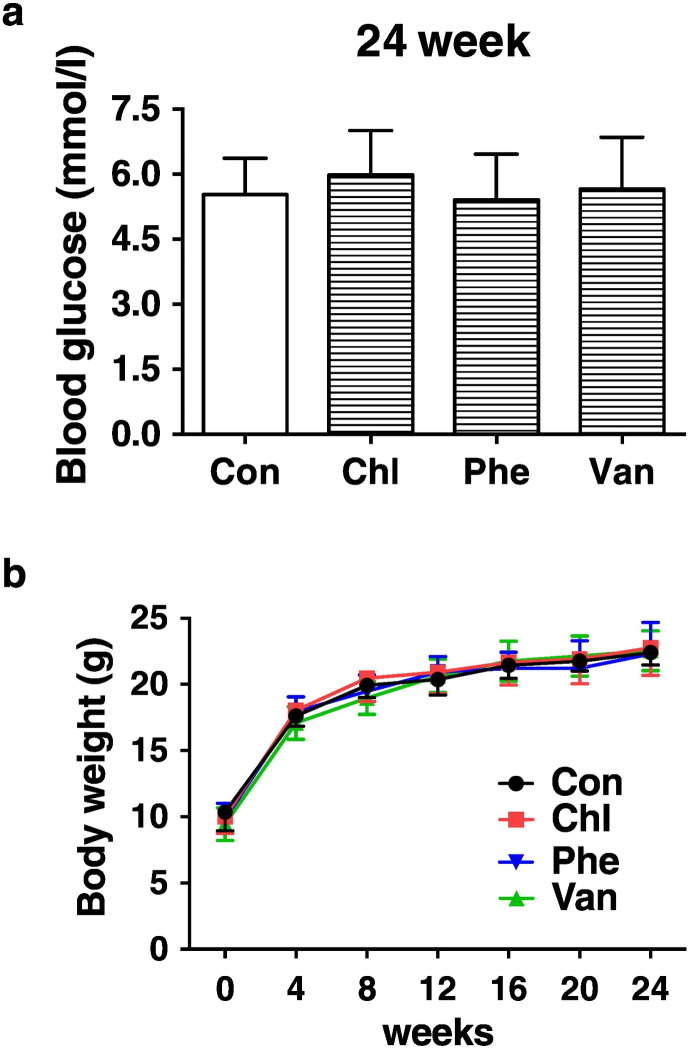
Effects of chronic subtherapeutic antibiotic exposure on blood glucose levels (a) and body weights (b) in mice. Throughout, error bars represent mean ± S.D. *n* = 10 per group. Con, control; Chl, chlortetracycline; Phe, penicillin; Van, vancomycin.

**Fig. 2:**
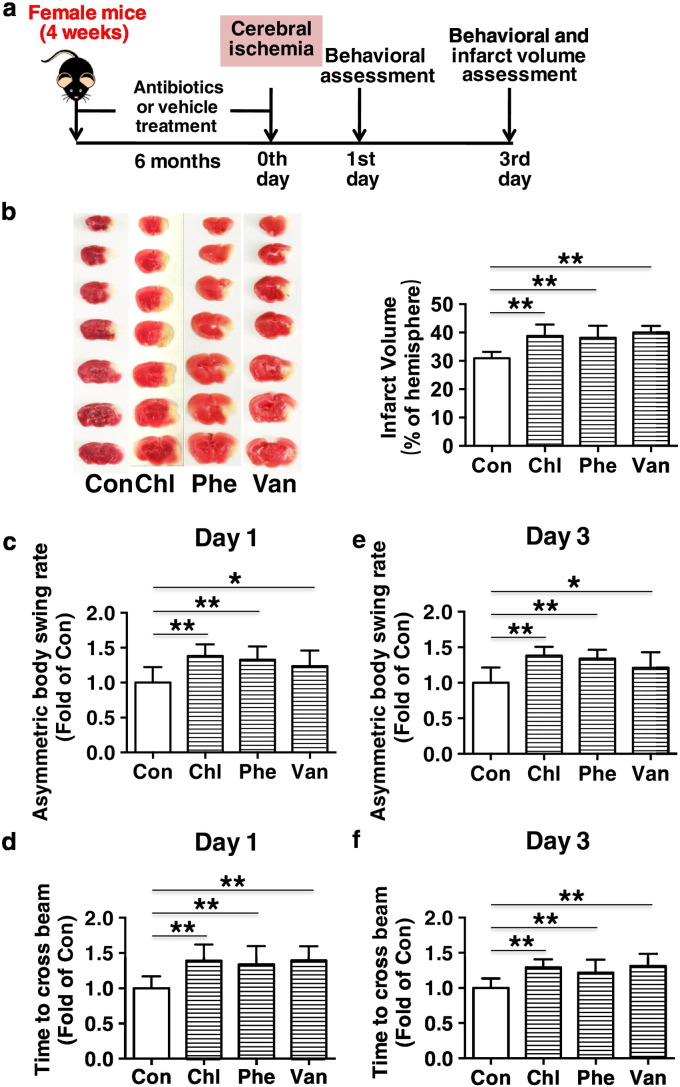
Chronic subtherapeutic antibiotic exposure aggravated cerebral ischemic injury in mice. (a) Surgical protocols: female C57BL/6 mice (4 weeks) were randomly allocated to 4 groups and respectively treated with chlortetracycline, penicillin, vancomycin or vehicle for 6 months. Then the mice were subjected to permanent focal cerebral ischemia. Behavioral assessment was performed at 1 and 3 days after cerebral ischemia, and then the infarct volumes were determined. (b) The representative images of TTC-stained brain sections (left) and cerebral infarct volumes (right). (c–f) Neurobehavioral outcomes (c and e, Body Asymmetry Test; d and f, Beam Test). Throughout, error bars represent mean ± S.D. Values were normalized to control. *P < 0.05, **P < 0.01. *n* = 10 per group. Con, control; Chl, chlortetracycline; Phe, penicillin; Van, vancomycin.

**Fig. 3:**
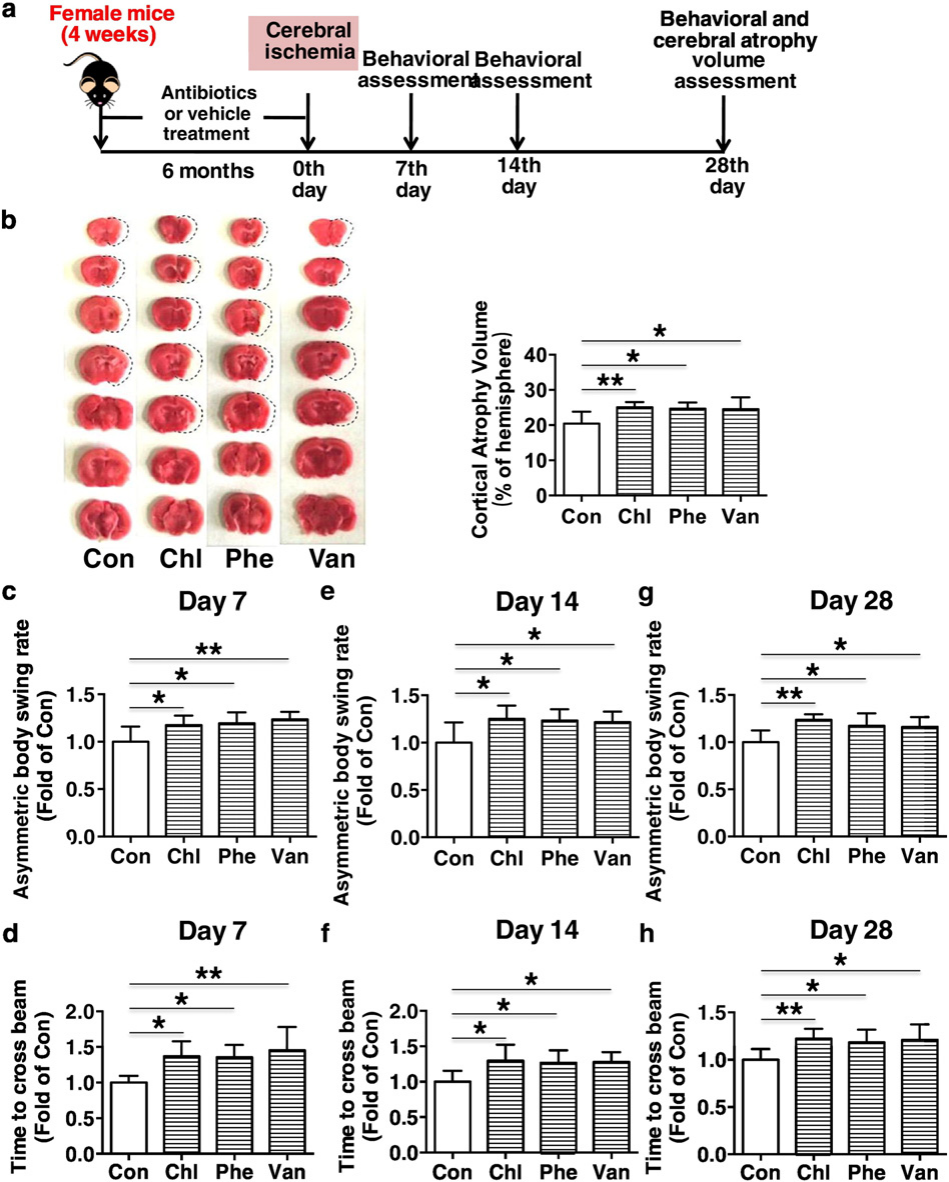
Chronic subtherapeutic antibiotic exposure before ischemia onset aggravated cortical atrophy and reduced long-term neurobehavioral outcomes after cerebral ischemia in mice. (a) Surgical protocols: female C57BL/6 mice (4 weeks) were randomly allocated to 4 groups and respectively treated with chlortetracycline, penicillin, vancomycin or vehicle for 6 months. Then the mice were subjected to permanent focal cerebral ischemia. Behavioral assessment was performed at 7, 14 and 28 days after cerebral ischemia, and then the cortical atrophy volumes were determined. (b) TTC staining showed much larger atrophy in the brains of the chronic antibiotic-exposed mice than that of the control mice. Quantitative analysis showed that chronic subtherapeutic antibiotic exposure significantly aggravated the brain atrophy 28 days after cerebral ischemia compared with control. *P < 0.05, **P < 0.01. *n* = 8 per group. (c–h) chronic subtherapeutic antibiotic exposure reduced neurobehavioral outcomes (c, e and g, Body Asymmetry Test; d, f and h, Beam Test) in mice. Values were normalized to Con. *P < 0.05, **P < 0.01. *n* = 8 per group. Throughout, error bars represent mean ± S.D. Con, control; Chl, chlortetracycline; Phe, penicillin; Van, vancomycin.

**Fig. 4:**
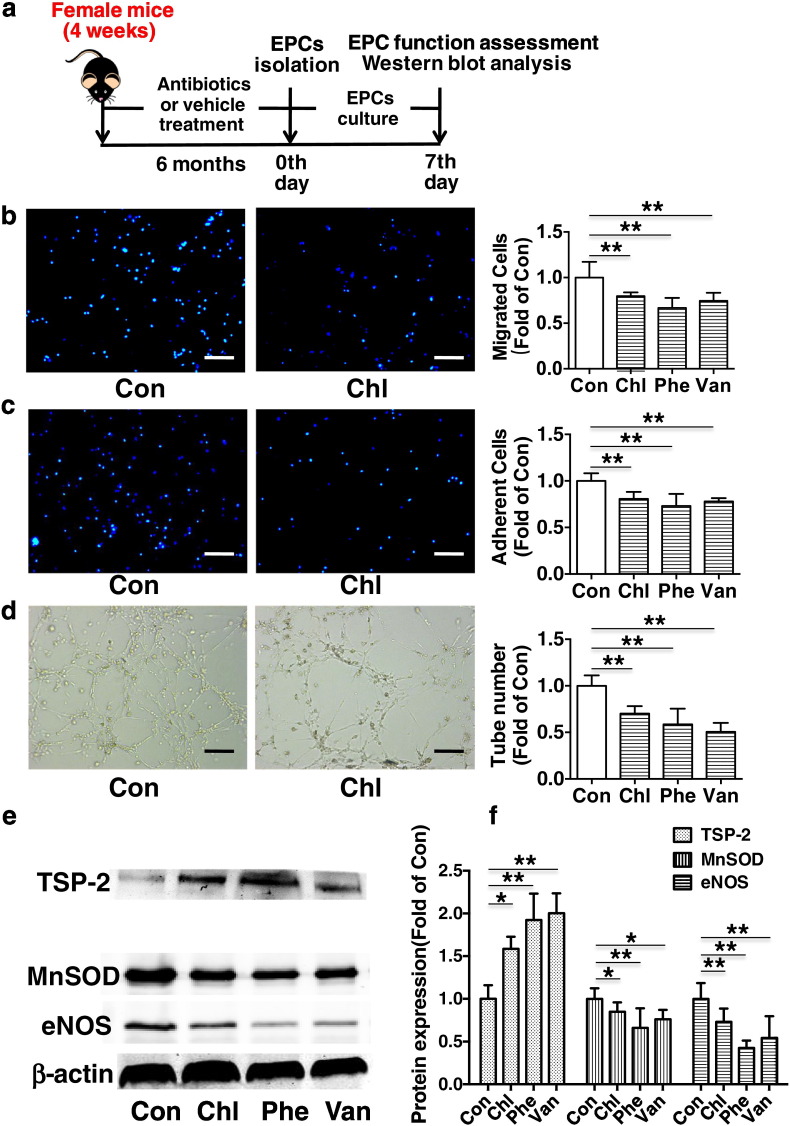
Chronic subtherapeutic antibiotic exposure impaired EPC functions in mice. (a) Surgical protocols: female C57BL/6 mice (4 weeks) were randomly allocated to 4 groups and respectively treated with chlortetracycline, penicillin, vancomycin or vehicle for 6 months. Then, the EPCs were isolated, cultured and examined in mice. (b) Migration assay of EPCs (*n* = 6). (c) Adhesion assay of EPCs (*n* = 6). (d) Tube formation assay of EPCs (*n* = 6). (e and f) The representative images and the protein expression levels of MnSOD (*n* = 8), eNOS (*n* = 8) and TSP-2 (*n* = 3). Throughout, error bars represent mean ± S.D. Values were normalized to control. *P < 0.05, **P < 0.01. Con, control; Chl, chlortetracycline; Phe, penicillin; Van, vancomycin. Scale bar: 100 μm.

**Fig. 5:**
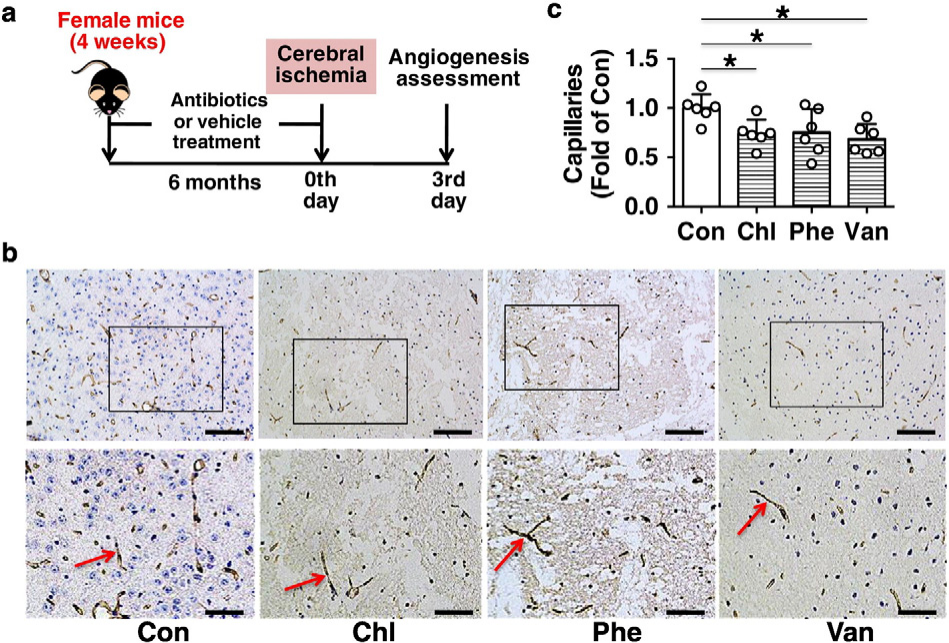
Chronic subtherapeutic antibiotic exposure reduced local angiogenesis in ischemic brain in mice. (a) Surgical protocols: female C57BL/6 mice (4 weeks) were randomly allocated to 4 groups and respectively treated with chlortetracycline, penicillin, vancomycin or vehicle for 6 months. Then the mice were subjected to permanent focal cerebral ischemia, and the local angiogenesis in ischemic brain was determined at 3 days after cerebral ischemia. (b) CD31 immunostaining shows microvessels in the ischemic boundary area of ischemic brains. (c) The bar graph shows that the number of microvessels in antibiotics-treated mice was significantly reduced compared with control. Throughout, error bars represent mean ± S.D. Values were normalized to control. *P < 0.05. *n* = 6 per group. Scale bars: 100 μm (top); 50 μm (bottom). Con, control; Chl, chlortetracycline; Phe, penicillin; Van, vancomycin.

**Fig. 6:**
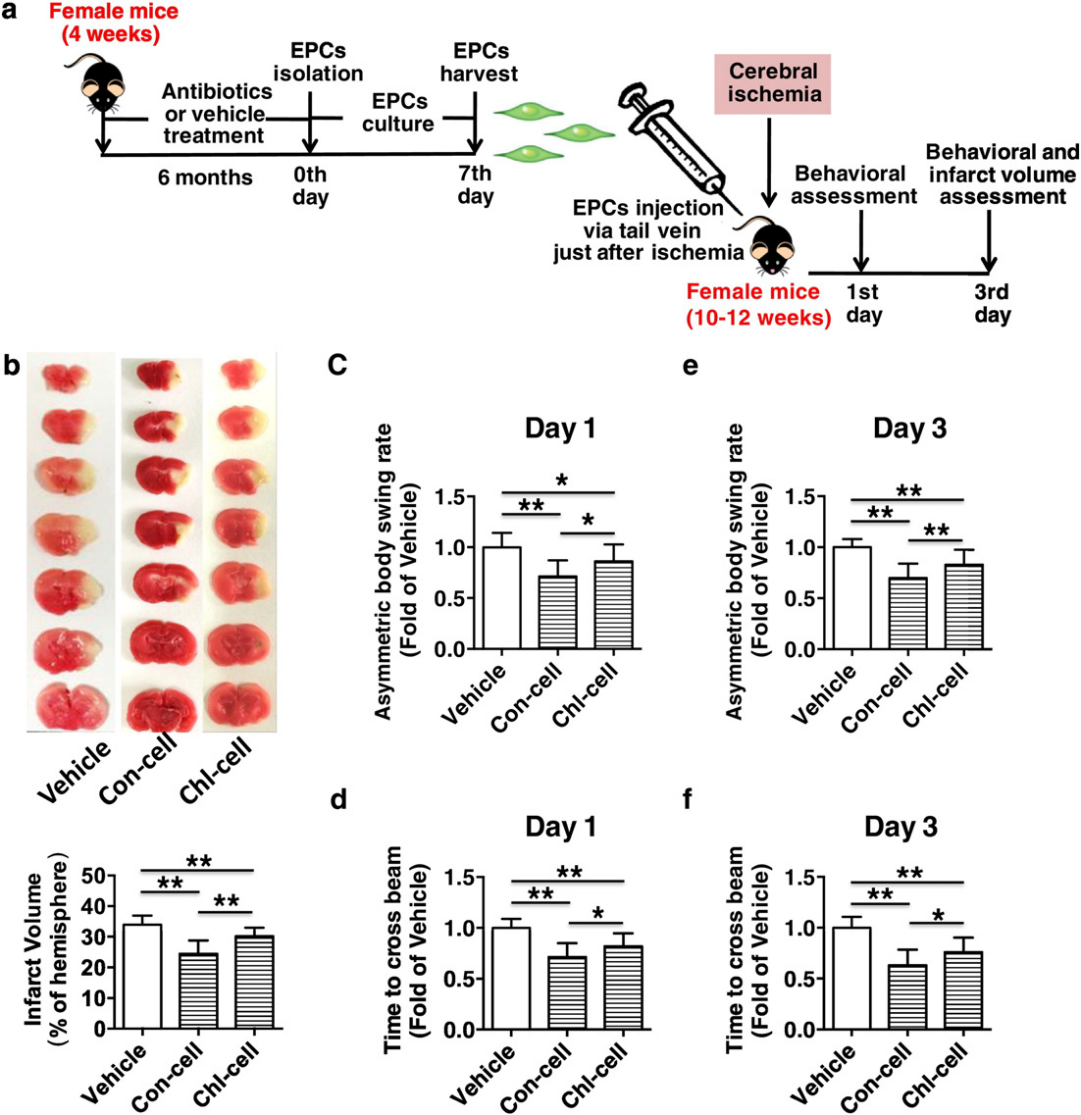
Chronic subtherapeutic antibiotic exposure reduced the therapeutic effect of EPCs on cerebral ischemic injury in mice. (a) Surgical protocols: female C57BL/6 mice (10–12 weeks) were randomly allocated to 3 groups. Each group of animals respectively received a single injection of vehicle, EPCs from control mice or EPCs from chronic antibiotic-exposed mice just after cerebral ischemia. Behavioral assessment was performed at 1 and 3 days after cerebral ischemia, and then the cerebral infarct volumes were determined. (b) The representative images of TTC-stained brain sections (top) and cerebral infarct volumes (bottom). (c–f) Neurobehavioral outcomes (c and e, Body Asymmetry Test; d and f, Beam Test). Throughout, error bars represent mean ± S.D. Values were normalized to control. *P < 0.05, **P < 0.01. *n* = 13–15 per group. Vehicle, mice treated with vehicle; Con-cell, mice treated with EPCs from control mice; Chl-cell, mice treated with EPCs from chronic chlortetracycline-exposed mice.

**Fig. 7:**
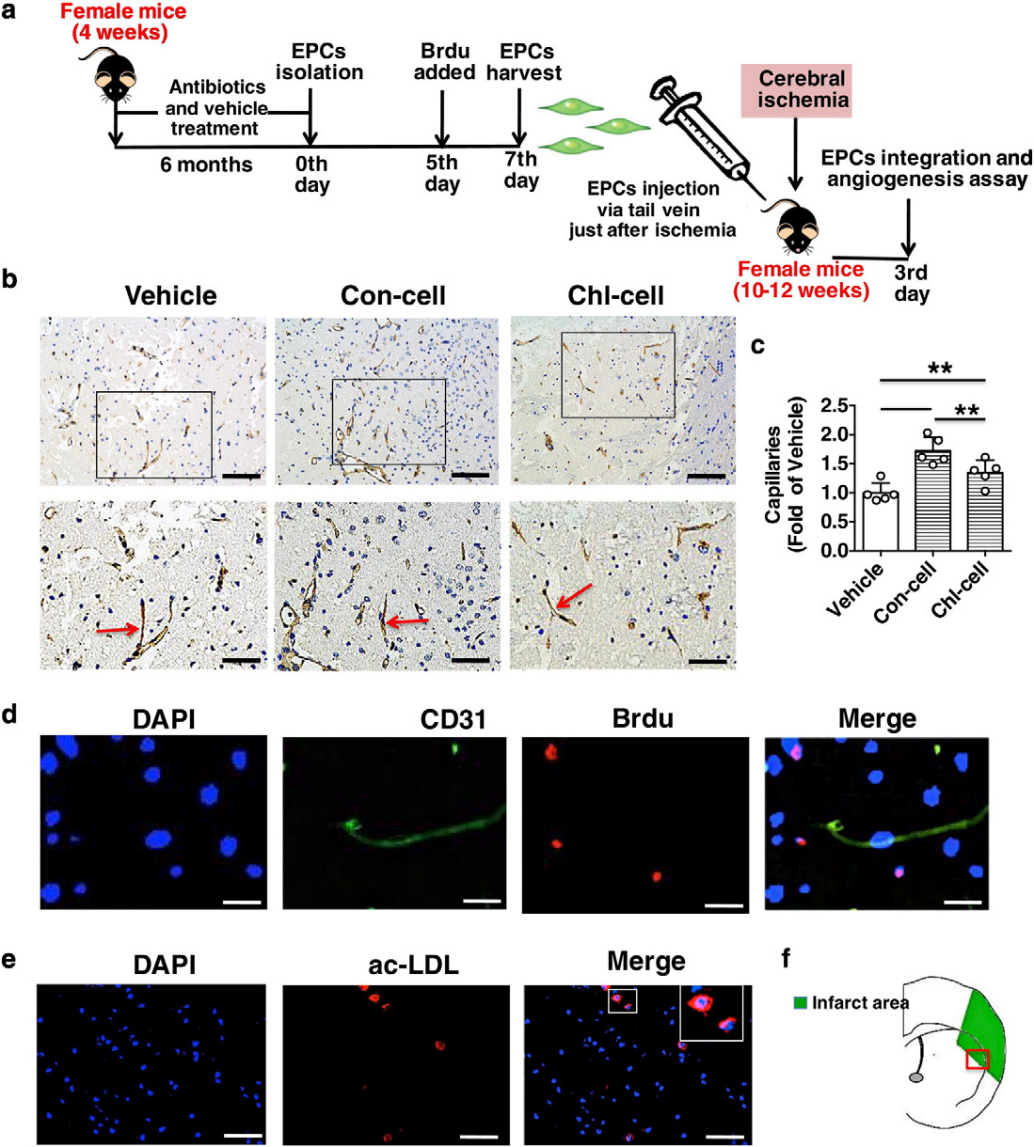
Chronic subtherapeutic antibiotic exposure reduced the therapeutic effect of EPCs on angiogenesis promotion in mice. (a) Surgical protocols: female C57BL/6 mice (10–12 weeks) were randomly allocated to 3 groups. Each group of animals respectively received a single injection of vehicle, EPCs from control mice or EPCs from chronic antibiotic-exposed mice just after cerebral ischemia. The local angiogenesis in ischemic brain were determined at 3 days after cerebral ischemia. (b) CD31 immunostaining shows microvessels in the ischemic boundary area of ischemic brains. (c) The bar graph shows that capillary density was significantly higher in the two groups of EPCs-treated mice compared with control, but the EPCs from chronic chlortetracycline-treated mice exerted a significantly lower effect on angiogenesis promotion compared to the EPCs from control mice. Throughout, error bars represent mean ± S.D. Values were normalized to control. *P < 0.05; **P < 0.01. *n* = 5 per group. Scale bars: 100 μm (top); 50 μm (bottom). (d) Typical photographs (taken from the ischemic boundary area) indicate that some BrdU-positive cells (red fluorescence) migrated into the ischemic area of brains and were found near the CD31-positive vessels (green fluorescence) after 3 days of injection. The nucleus was counterstained with DAPI (blue fluorescence). Scale bar: 20 μm. (e) Photomicrographs (taken from the ischemic boundary area) show that Dil-labeled EPCs (red fluorescence) migrated into the ischemic area of brains after 3 days of injection. The nucleus was counterstained with DAPI (blue fluorescence). Inserted boxes show increased magnification of cells. Scale bar: 50 μm. (f) The figure shows the ischemic area and the ischemic boundary area of ischemic brain. Vehicle, mice treated with vehicle; Con-cell, mice treated with EPCs from control mice; Chl-cell, mice treated with EPCs from chronic chlortetracycline-exposed mice.

**Fig. 8:**
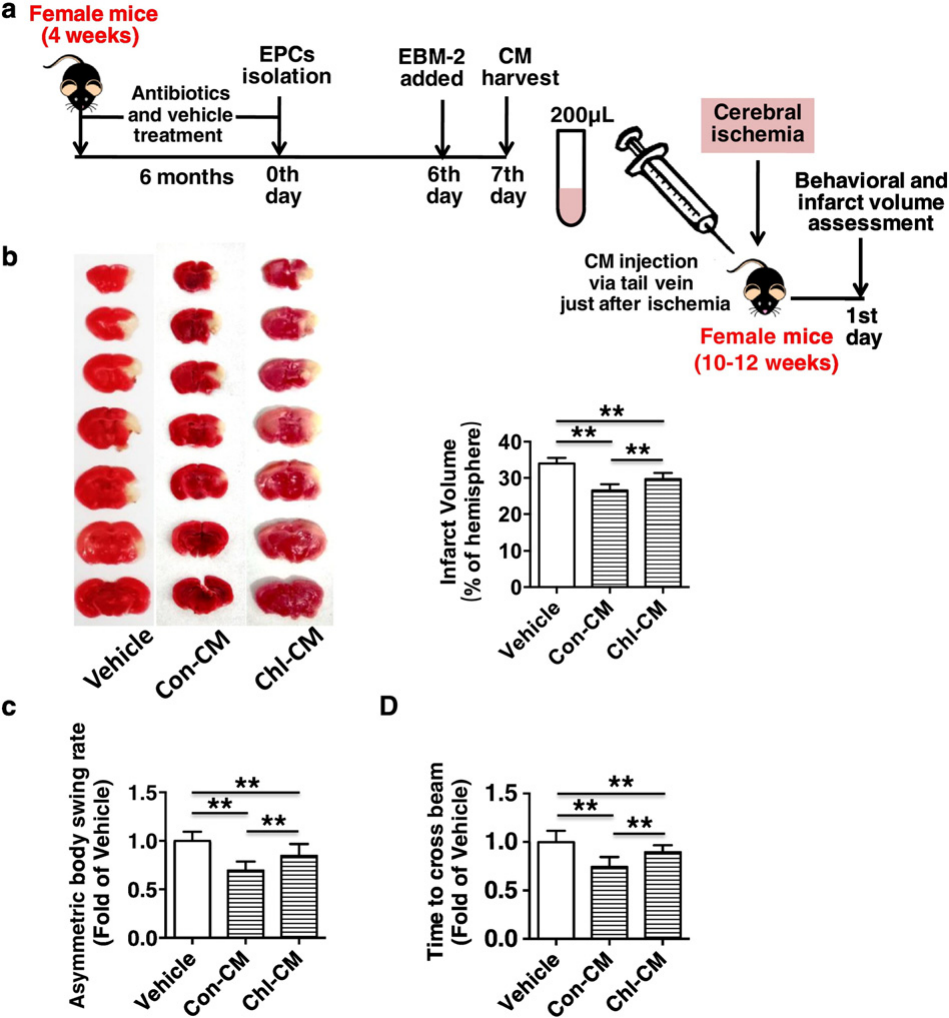
Chronic subtherapeutic antibiotic exposure reduced the therapeutic effect of EPC-conditioned culture media (CM) on cerebral ischemic injury in mice. (a) Surgical protocols: female C57BL/6 mice (10–12 weeks) were randomly allocated to 3 groups. Each group of animals respectively received a single injection of vehicle, CM from control mice or CM from chronic antibiotic-exposed mice just after cerebral ischemia. Behavioral tests were performed at 1 day after cerebral ischemia, and then the cerebral infarct volumes were determined. (b) The representative images of TTC-stained brain sections (left) and cerebral infarct volumes (right). (c) and (d) Neurobehavioral outcomes (c, Body Asymmetry Test; d, Beam Test). Throughout, error bars represent mean ± S.D. Values were normalized to control. *P < 0.05; **P < 0.01. *n* = 8–10 per group. Vehicle, mice treated with vehicle; Con-CM, mice treated with CM from control mice; CM-Cold, mice treated with CM from chronic chlortetracycline-exposed mice.

**Fig. 9:**
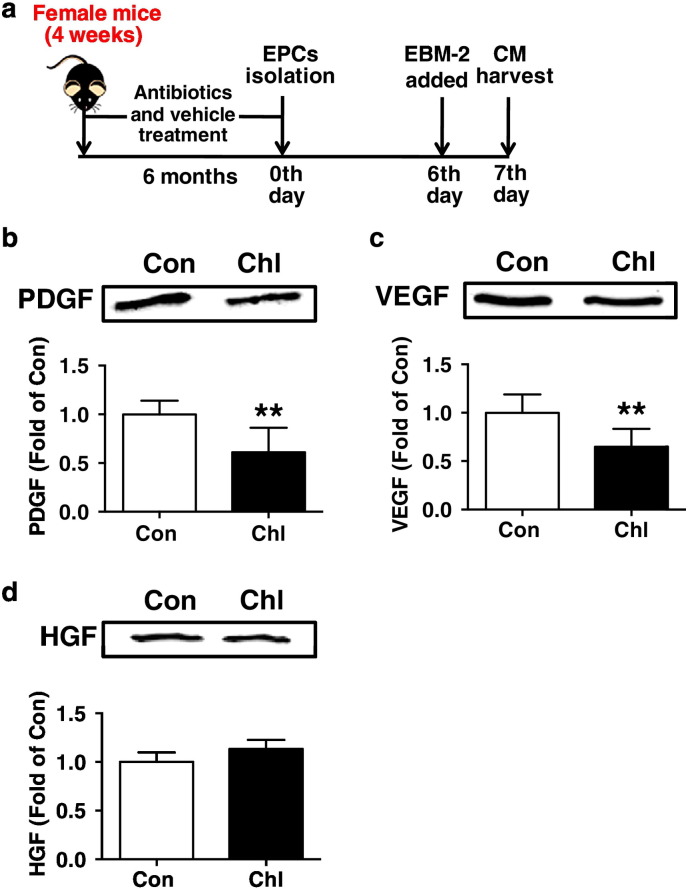
Effects of chronic subtherapeutic antibiotic exposure on secreted PDGF, VEGF and HGF levels in EPC-conditioned culture media (CM) in mice. (a) Surgical protocols: female C57BL/6 mice (4 weeks) were randomly allocated to 2 groups and respectively treated with chlortetracycline or vehicle for 6 months. Then, the EPCs were isolated and cultured, and EPC-conditioned culture media was collected and concentrated. Secreted PDGF, VEGF and HGF levels in CM were assessed by Western blot analysis. (b) Secreted PDGF levels in CM (*n* = 7). (c) Secreted VEGF levels in CM (*n* = 6). (d) Secreted FGF levels in CM (*n* = 5). Throughout, error bars represent mean ± S.D. Values were normalized to control. **P < 0.01 vs. Con. Con, control; Chl, chlortetracycline; PDGF, platelet-derived growth factor; VEGF, vascular endothelial growth factor; HGF, hepatocyte growth factor.

In our preliminary study, it was found that, beginning at 4 weeks of age, exposure to the aforementioned antibiotics via drinking water (1 μg antibiotic per g body weight of mice) for 6 weeks did not significantly impaired EPC functions and aggravated cerebral ischemic injury in mice (data not shown). Therefore, we extended the antibiotic exposure period to 6 month in the present work (Fig. 1, Fig. 2, Fig. 3, Fig. 4, Fig. 5, Fig. 6, Fig. 7, Fig. 8, Fig. 9).

After 6 months of treatment, mice were subjected to permanent focal cerebral ischemia (Figs. 2a and 3a), or were used for EPCs isolation and assessment (Fig. 4a). Administration of antibiotics was discontinued 3 day before EPCs isolation or cerebral ischemia to avoid off-target effects of antibiotics.

### 2.2 Chronic Subtherapeutic Antibiotic Exposure and Animal Model of Stroke

After 6 months of treatment, the mice were subjected to permanent focal cerebral ischemia according to published protocols and our previous work (Dong et al., 2015; Zhu et al., 2008). Mice were anesthetized by intraperitoneal injection of 0.1 ml 3.5% chloral hydrate per 10 g body weight. A skin incision was made between the ear and the orbit on the left side. After splitting of the temporalis muscle, a burr hole was drilled at the junction of the zygomatic arch and the squamous bone, through which the stem of the left middle cerebral artery (MCA) was exposed and occluded by electrocoagulation. Body temperature was maintained at 37 ± 0.5 °C using a thermal blanket throughout the surgical procedure. The regional cerebral blood flow (rCBF) was monitored by Laser-Doppler flowmetry (moorVMS-LDF1, Moor Instruments Ltd., England) before and after left middle cerebral artery occlusion. Mice with regional cerebral blood flow that was > 15% of the baseline were excluded from the experiment. Behavioral tests (including Body Asymmetry Test and Beam Test) were performed 1 and 3 days after MCA occlusion, and then animals were euthanized and the brains were stained with 2,3,5-triphenyltetrazolium chloride (TTC) (Sigma, USA) to determine the infarct volume, which was corrected for swelling/edema, as previously reported (Fig. 2a, *n* = 10 per group) (Dong et al., 2015; Zhu et al., 2008).

After 3 days of cerebral ischemia, the mice (Fig. 5a, *n* = 6 per group) were euthanized and the ischemic brains were fixed by transcardial perfusion with saline, followed by perfusion and immersion in 4% paraformaldehyde, before being embedded in paraffin. A series of 6-μm-thick sections was cut from the block. Every 10th coronal section for a total three sections was used for immunohistochemical staining. Antibody against CD31 (BD Biosciences) immunostaining was performed to detect the angiogenesis in the ischemic brain (Dong et al., 2015; Zhang et al., 2002; Chen et al., 2005).

Furthermore, as showed in Fig. 3a (*n* = 8 per group), long-term neurobehavioral outcomes of mice were assessed at 7, 14 and 28 days after cerebral ischemia (Dong et al., 2015; Fan et al., 2010; Zhu et al., 2008). After 28 days of cerebral ischemia, animals were euthanized and the brains were stained with 2,3,5-triphenyltetrazolium chloride (TTC) to determine the cerebral atrophy volumes as previously reported (Fan et al., 2010). Cerebral atrophy volume was calculated by subtraction of the volume of ipsilateral hemisphere from the volume of contralateral hemisphere.

### 2.3 Neurological Behavior Analysis: Body Asymmetry Test and Beam Test

Body Asymmetry Test – Asymmetric motor behavior was quantitatively analyzed with the use of the elevated body swing test as previously described (Dong et al., 2015; Fan et al., 2010). Briefly, mice were examined for lateral turning when their bodies were suspended by lifting their tails. The frequency of initial turning of head contralateral to the ischemic side was counted in 20 consecutive trials and was normalized as follows: (Number of lateral turns in 20 trials − 10)/10 × 100%.

Beam Test – Beam walking across a bridge was used to assess motor coordination and balance ability after stroke injury (Dong et al., 2015; Fan et al., 2010). Mice were trained for 5 days before MCA occlusion to traverse a round beam (5 mm diameter and 900 mm in length) to reach an enclosed escape platform. They were placed on one end of the beam and the latency to traverse the 80% of the beam toward the enclosed escape platform at the other end was recorded. Data are expressed as mean latency to cross the beam of 3 trials.

### 2.4 Bone Marrow-derived EPCs Isolation and Culture

After 6 months of treatment, bone marrow-derived mononuclear cells (BM-MNCs) were isolated from mice (Fig. 4a) tibia and femur as described previously (Dong et al., 2015; Marrotte et al., 2010; Xie et al., 2010). Cells were seeded in 6-well cell culture plates coated with rat vitronectin (Sigma) at a density of 5 × 10^6^ cells/well, and cultured in endothelial growth medium-2 (EGM-2, Lonza). After 4 days of culture, nonadherent cells were removed, and the adherent cells were further cultivated for 3 days. The cells were then used for in vitro studies (including function assays and Western blot analysis) (Fig. 4a), EPC transplantation (Figs. 6a and 7a) and EPCs' paracrine effect assessment (Figs. 8a and 9a).

### 2.5 In Vitro Cell Function Assays

#### 2.5.1 Migration Assay

A number of 5 × 10^4^ cells were applied into upper Boyden's chamber with M199. The lower chambers were loaded with M199 supplemented 50 ng/ml VEGF. After 24 h, migrated cells were fixed and stained with Hoechst 33258 (10 μg/ml, Sigma, America). The number of cells on the lower side of the membrane was counted at magnification × 100, and the mean value of 5 different areas was determined for each sample (Dong et al., 2015).

#### 2.5.2 Adhesion Assay

1 × 10^4^ EPCs were allowed to adhere to 96-well plates coated with 1 μg/ml vitronectin (Sigma-Aldrich) for 4 h. After washing 3 times with PBS, residual adherent cells were stained with Hoechst 33258 for 15 min and fixed with 2% paraformaldehyde. The number of adherent cells was counted at random in 5 high-power fields (magnifications × 100) per sample, and the mean value of the three wells was determined for each sample (Dong et al., 2015; Xie et al., 2010).

#### 2.5.3 Tube Formation Assay

Growth factor-reduced Matrigel-Matrix (BD Biosciences) was placed in a 96-well cell culture plate at 37 °C, 5% CO_2_ for 30 min. 5 × 10^4^ EPCs were plated in each well. After 6 h of incubation, images of tube morphology was taken by inverted microscope (Nikon) and tube numbers were counted at random under 5 high-power fields (magnifications × 100) per sample (Dong et al., 2015; Marrotte et al., 2010; Xie et al., 2010).

### 2.6 Western Blot Analysis

Western blot analysis was performed as we previously described (Marrotte et al., 2010; Xie et al., 2010). Briefly, collected EPC culture media were concentrated with an Amicon Ultra 4 centrifugal filter device with a 10,000 molecular weight cutoff (Millipore) according to the manufacturer's recommendations. Protein concentrations were determined using the BCA protein assay kit (Pierce, Thermo), and samples containing equal amounts of protein were subjected to 8% SDS/PAGE. Gels were transferred to nitrocellulose membranes and incubated with mouse anti-TSP-2 (Abcam Inc.) monoclonal antibody. Secondary antibodies included IR Dye 800 conjugated anti-mouse IgG (1:5000, Rockland). Bands were visualized using Odyssey Imager with Odyssey 1.1 software (Li-Cor) and quantified using NIH ImageJ software.

EPCs were washed with cold PBS twice and then incubated in lysis buffer. The BCA protein assay kit (Pierce, Thermo) was used to quantify protein. Proteins of equal quantities were subjected to 8–10% SDS/PAGE gels. Subsequently, they were transferred to nitrocellulose membranes. The membranes were blocked for 0.5 h in 5% milk in phosphate buffer saline (PBS). Then washed the membranes three times for 5 min and incubated with primary antibodies which were purified mouse anti-eNOS (BD Transduction Labs) and mouse anti-MnSOD (BD Transduction Labs) at a dilution of 1:1000 overnight. Washed the membranes three times with PBST (containing 0.1% Tween-20), and bound antibody was detected using anti-mouse IgG secondary antibody (1:5000, CST) (eNOS and MnSOD). Bands were visualized using Odyssey Imager with Odyssey 1.1 software (Li-Cor) and quantified using NIH image J software.

### 2.7 EPC Transplantation and Animal Stroke Model in Mice

To further determine whether the EPC-mediated angiogenesis is involved in cerebral ischemic injury aggravation produced by chronic exposure to subtherapeutic antibiotics in mice, 1 × 10^6^ bone marrow-derived EPCs in 200 μl PBS from chronic chlortetracycline-treated and control mice were systemically administrated to female C57BL/6 mice (10–12 weeks, Sino-British SIPPR/BK Lab Animal Ltd., Shanghai, China.) via the tail vein just after cerebral ischemia, and control animals received equal volume of vehicle (Fig. 6a, n = 13–15 per group) (Fan et al., 2010; Zhang et al., 2002). At 1 and 3 days after cerebral ischemia, behavioral tests (including Body Asymmetry Test and Beam Test) were performed, and then the brains were stained with 2,3,5-triphenyltetrazolium chloride (TTC) to determine the infarct volume as previously reported (Dong et al., 2015; Zhu et al., 2008).

After 3 days of cerebral ischemia, the mice (Fig. 7a, n = 5 per group) were euthanized and the ischemic brains were fixed by transcardial perfusion with saline, followed by perfusion and immersion in 4% paraformaldehyde, before being embedded in paraffin. A series of 6-μm-thick sections was cut from the block. Every 10th coronal section for a total three sections was used for immunohistochemical staining. Antibody against CD31 (BD Biosciences) immunostaining was performed to detect the angiogenesis in the ischemic brain (Dong et al., 2015; Zhang et al., 2002; Chen et al., 2005).

### 2.8 EPC Transplantation and In Vivo EPC Integration in Mice

As showed in Fig. 7a, on day 5 of culture, EPCs from chronic chlortetracycline-treated and control mice were labeled with 5-bromo-2′-deoxyuridine (BrdU, Thermo Fisher SCIENTIFIC), as described previously (Marrotte et al., 2010). Briefly, BrdU-labeling reagent was diluted to 1:100 in EGM-2, filtered through a 0.2-μm filter, and warmed to 37 °C. 2 ml of BrdU/EGM-2 was added to cells in a 6-well plate, and new media were added daily until day 7. On day 7, the wells were washed 3 times with PBS, followed by trypsinization to resuspend the cells. Mouse EPCs (1 × 10^6^ cells) were then respectively transplanted into female C57BL/6 mice (10–12 weeks) via the tail vein just after cerebral ischemia.

After 3 days of cerebral ischemia, the mice were euthanized and the ischemic brains were fixed by transcardial perfusion with saline, followed by perfusion and immersion in 4% paraformaldehyde, before being embedded in paraffin. A series of 6-μm-thick sections were cut from the block. Every 10th coronal section for a total three sections was used for immunohistochemical staining (Dong et al., 2015; Zhang et al., 2002; Chen et al., 2005). To detect the in vivo EPC integration, slides were stained with anti-CD31 antibody (BD Biosciences), followed by BrdU antibody (Santa Cruz Bio-technology Inc.) incubation. The secondary antibodies were AlexaFluor 488 (Abcam) or Cy3 (Abcam). The nucleus was counterstained with DAPI (Cell Signaling Technology) (Fan et al., 2010; Marrotte et al., 2010).

In addition, Dil-labeled EPCs were also used for cell tracking in ischemic brain to detect the in vivo EPC integration in mice. After 7 days of culture, EPCs from were extensively washed with PBS and stained with 2.5 μg/ml of Dil-labeled acetylated low-density lipoprotein (Invitrogen) at 37 °C for 60 min. Dil-labeled EPCs (1 × 10^6^ cells) in 200 μl PBS were transplanted into female C57BL/6 mice (10–12 weeks) via the tail vein just after cerebral ischemia (Fan et al., 2010; Zhang et al., 2002). After 3 days of cerebral ischemia, the mice were euthanized and the ischemic brains were harvested. The cell nucleus was counterstained with DAPI (Cell Signaling Technology), and the Dil-labeled EPCs were detected in frozen ischemic brain sections (Fan et al., 2010).

### 2.9 Treatment of Cerebral Ischemic Injury with EPC-conditioned Media in Mice

As described previously (Marrotte et al., 2010; Rosell et al., 2013), bone marrow-derived mononuclear cells from chronic chlortetracycline-treated and control mice were plated on a vitronectin (Sigma-Aldrich)-coated 6-well plate at 5 × 10^6^ cells/well in EGM-2 at 37 °C with 5% CO_2_. After 4 days of culture, nonadherent cells were removed and fresh EGM-2 was changed daily. At day 6 of culture, growing EPCs were washed twice with endothelial basal medium-2 without growth factors and serum (EBM-2, Lonza). Then, the fresh EBM-2 (1.5 ml/well) was added to obtain conditioned media (CM) that was collected 24 h later. 4 ml CM was concentrated for 40 min using a 10 kDa filter unit (Millipore, Ireland), for a final volume of approximately 200 μl. As showed in Fig. 8a (*n* = 8–10 per group), the concentrated CM (200 μl) was injected into female C57BL/6 mice (10–12 weeks) via the tail vein just after cerebral ischemia, and control mice received equal volume of vehicle (EBM-2) (Marrotte et al., 2010; Rosell et al., 2013). After 24 h of cerebral ischemia, behavioral tests (including Body Asymmetry Test and Beam Test) were performed and cerebral infarct volumes were determined as previously reported (Dong et al., 2015; Zhu et al., 2008).

In addition, to investigate the potential effect of chronic subtherapeutic antibiotic exposure on paracrine factors of EPCs in mice, the secreted platelet-derived growth factor (PDGF), vascular endothelial growth factor (VEGF) and hepatocyte growth factor (HGF) levels in the concentrated CM were assessed by Western blot analysis (Marrotte et al., 2010; Rosell et al., 2013; Xie et al., 2010) (Fig. 9a). Protein concentrations were determined using the BCA protein assay kit (Pierce, Thermo), and samples containing equal amounts of protein were subjected to 8% SDS/PAGE. Gels were transferred to nitrocellulose membranes and incubated with rabbit anti-PDGF polyclonal antibody (Abcam Inc.), rabbit anti-VEGF polyclonal antibody (Abcam Inc.) and rabbit anti-HGF polyclonal antibody (Abcam Inc.). Secondary antibody was IR Dye 800 conjugated anti-rabbit IgG (1:2000, Rockland). Bands were visualized using Odyssey Imager with Odyssey 1.1 software (Li-Cor) and quantified using NIH image J software.

### 2.10 Statistical Analysis

Data are expressed as mean ± S.D. Statistical significance of difference between two groups was performed using the Student's unpaired *t*-test. When more than two groups were compared, one-way ANOVA followed by Tukey post hoc analysis was used. A value of *P* < 0.05 was considered statistically significant.

## 3 Results

### 3.1 Chronic Subtherapeutic Antibiotic Exposure Showed No Effect on Fasting Blood Glucose Levels and Body Weights in Mice

We exposed C57BL/6 mice at weaning to chlortetracycline, penicillin, vancomycin, or no antibiotic in their drinking water at levels in the mid-range of FDA-approved levels for subtherapeutic antibiotic use in agriculture (Butaye et al., 2003; Cho et al., 2012). It was found that there was no significant difference in fasting blood glucose levels among all the groups of mice before (data not shown) or after 6 months of treatment (Fig. 1a). Previous study demonstrated that subtherapeutic antibiotic exposure for 7 weeks increased fat mass but did not affect the body weights in mice (Cho et al., 2012). The present work showed that no significant difference in body weights was found among all the groups of mice before or during 6 months of treatment (Fig. 1b).

### 3.2 Chronic Subtherapeutic Antibiotic Exposure Aggravated Cerebral Ischemic Injury in Mice

After 6 months of treatment, mice were subjected to permanent focal cerebral ischemia, and 3 days later, their cerebral ischemic injury was determined (Fig. 2a). It was found that the infarct volumes were significantly increased (+ 25.5%, *p* < 0.01; + 23.3%, *p* < 0.01 or + 29.4%, *p* < 0.01 respectively) and the corresponding neurobehavioral outcomes were markedly impaired in chronic chlortetracycline, penicillin or vancomycin-treated mice compared with control (Fig. 2b to f). These results suggest that long-term exposure to these subtherapeutic antibiotics was able to aggravate cerebral ischemic injury in mice.

### 3.3 Chronic Subtherapeutic Antibiotic Exposure Aggravated Brain Cortex Atrophy and Reduced Long-Term Neurobehavioral Outcomes after Cerebral Ischemia in Mice

We further evaluated the potential long-term effects of chronic subtherapeutic antibiotic exposure on cerebral ischemic injury (Fig. 3a). We found that, at 28 days after cerebral ischemia, the overall atrophy volumes of hemisphere in chronic chlortetracycline, penicillin or vancomycin-treated mice were significantly increased (*p* < 0.05) compared to control (Fig. 3b). We also examined the neurobehavioral outcomes at 7, 14 and 28 days after cerebral ischemia, and it was found that neurobehavioral outcomes (both Beam Test and Body Asymmetry Test) were markedly reduced in chronic antibiotics-exposed mice compared with control (Fig. 3c to h). These results indicate that chronic exposure to these subtherapeutic antibiotics could reduce long-term neurobehavioral outcome after cerebral ischemia in mice.

### 3.4 Chronic Subtherapeutic Antibiotic Exposure Impaired EPC Functions and Reduced Local Angiogenesis in Ischemic Brain in Mice

To investigate the possible mechanisms underlying chronic antibiotic exposure aggravating cerebral ischemic injury in mice, the EPC functions were determined in mice after 6 months of treatment (Fig. 4a). It was found that EPC functions (including migration, tube formation and adhesion function) were significantly impaired in chronic antibiotics-treated mice compared with control (Fig. 4b to d).

To determine the potential mechanisms underlying EPC function impairment in chronic antibiotics-exposed mice, MnSOD, eNOS and thrombospondin-2 (TSP-2) expression levels were examined (Fig. 4a). We found that both MnSOD and eNOS expression levels were significantly reduced in EPCs from chronic antibiotics-exposed mice compared to control. In addition, TSP-2 expression levels were markedly up-regulated in EPCs from chronic antibiotics-exposed mice compared to control (Fig. 4e,f).

In addition, EPCs can secret various angiogenic growth factors to promote ischemic angiogenesis (Fan et al., 2010). Thus, angiogenesis in ischemic brain was assessed at 3 days after cerebral ischemia (Fig. 5a). It was found that capillary density was significantly lower in chronic chlortetracycline, penicillin or vancomycin-treated mice compared with control (− 25.9%, *p* < 0.05; − 24.5%, *p* < 0.05; or − 31.4%, *p* < 0.05 respectively) (Fig. 5b,c). These results suggest that long-term exposure to these subtherapeutic antibiotics was able to impair EPC functions and reduce local angiogenesis in ischemic brain in mice.

### 3.5 Chronic Subtherapeutic Antibiotic Exposure Reduced the Therapeutic Effect of EPCs on Cerebral Ischemic Injury Reduction and Angiogenesis Promotion in Mice

To further determine whether the EPC-mediated angiogenesis is involved in cerebral ischemic injury aggravation produced by chronic exposure to subtherapeutic antibiotics in mice, 1 × 10^6^ bone marrow-derived EPCs from 6-month chlortetracycline-treated and control mice were respectively injected into mice via the tail vein just after cerebral ischemia, and control animals received equal volume of vehicle (Fig. 6a). As chronic treatment with subtherapeutic chlortetracycline, penicillin or vancomycin showed a similar effect on mouse EPC functions and cerebral ischemic injury in the present work, we used low-dose chlortetracycline as a model agent in the subsequent studies about EPC transplantation and EPC-conditioned media. It was found that the infarct volumes were significantly reduced (Con-cell: − 28.4%, *p* < 0.01 vs. Vehicle; Chl-cell: − 13.4%, *p* < 0.01 vs. Vehicle) and the corresponding neurobehavioral outcomes were markedly improved in the two groups of EPCs-treated mice compared with control. However, the EPCs from chronic chlortetracycline-treated mice exerted a significantly lower therapeutic effect on cerebral ischemic injury compared to the EPCs from control mice (*p* < 0.05) (Fig. 6b to f).

Furthermore, angiogenesis in ischemic brain was assessed at 3 days after cerebral ischemia (Fig. 7a). It was found that capillary density was significantly higher in the two groups of EPCs-treated mice compared with control (Con-cell: + 72.3%, *p* < 0.01 vs. Vehicle; Chl-cell: + 34.8%, *p* < 0.05 vs. Vehicle). However, the EPCs from chronic chlortetracycline-treated mice exerted a significantly lower effect on angiogenesis promotion compared to the EPCs from control mice (*p* < 0.05) (Fig. 7b,c).

To verify that BrdU-labeled EPCs were incorporated into the ischemic brains, the slides were stained with EC-specific marker CD31, followed by BrdU staining. As shown in Fig. 7d, some BrdU-positive cells (red fluorescence) migrated into the ischemic area and were found near the CD31-positive microvessels (green fluorescence) in the ischemic brain of mice that received EPC transplantation. In addition, Dil-labeled EPCs were also used for cell tracking in ischemic brain to detect the in vivo EPC integration in mice. It was found that intravenously delivered Dil-labeled EPCs (red fluorescence) could home into ischemic brain at 3 days after injection in mice (Fig. 7e). Previous studies have shown that transplanted EPCs can participate in ischemic brain angiogenesis or wound angiogenesis (Fan et al., 2010; Marrotte et al., 2010). The present findings suggested that injected EPCs integrated into ischemic brain and participated in angiogenesis in mice.

These results indicate that the transplanted EPCs could home to ischemic brain, promote local angiogenesis and protect against the cerebral ischemic injury in mice. However, chronic exposure to subtherapeutic chlortetracycline reduced the therapeutic effect of EPCs on angiogenesis promotion and cerebral ischemic injury reduction in mice.

### 3.6 EPC Transplantation Improved Neurobehavioral Outcomes after 1 Day of Cerebral Ischemia in Mice

It has been demonstrated that an angiogenic reaction starts between 24 and 48 h after cerebral infarction (Marti et al., 2000). To determine whether the transplanted EPCs may possess non-angiogenic effects (such as paracrine effects) on cerebral ischemic injury in addition to their direct angiogenic effects, we assessed the neurobehavioral outcomes at 24 h after cerebral ischemia in mice (Fig. 6a). After 1 day of cerebral ischemia, it was found that the neurobehavioral outcomes were significantly improved in the two groups of EPC-treated mice compared with control, and the EPCs from chronic chlortetracycline-treated mice exerted a significantly lower therapeutic effect on neurobehavioral outcome improvement compared to the EPCs from control mice (*p* < 0.05) (Fig. 6c,d).

These results indicate that transplanted EPCs might exert non-angiogenic effects on cerebral ischemic injury in addition to their direct angiogenic effects in mice.

### 3.7 Chronic Subtherapeutic Antibiotic Exposure Reduced the Therapeutic Effect of EPC-conditioned Culture Media on Cerebral Ischemic Injury in Mice

To study the paracrine effect of EPCs on cerebral ischemic injury, the conditioned media (CM) of mouse EPCs were collected and injected into mice via the tail vein (Fig. 8a). It was showed that the infarct volumes were significantly reduced (Con-CM: − 27.9%, *p* < 0.01 vs. Vehicle; Chl-CM: − 11.6%, *p* < 0.01 vs. Vehicle) and the corresponding neurobehavioral outcomes were markedly improved in the two groups of CM-treated mice compared with control. However, the CM of EPCs from chronic chlortetracycline-treated mice exerted a significantly lower therapeutic effect on cerebral ischemic injury compared to the CM of EPCs from control mice (*p* < 0.01) (Fig. 8b to d).

To investigate the potential effect of chronic subtherapeutic antibiotic exposure on paracrine factors of EPCs in mice, the secreted PDGF, VEGF and HGF levels in EPC-conditioned culture media were assessed (Fig. 9a). It was found that the secreted PDGF and VEGF levels were significantly reduced in EPC-CM from chronic chlortetracycline-treated mice compared with control (Fig. 9b,c). However, no significant difference in HGF levels in EPC-CM was found between these two groups of mice (Fig. 9d).

Previous studies have shown that EPC-conditioned media can accelerate diabetic wound healing (Marrotte et al., 2010), and an intravenous injection of EPC-conditioned media can enhance neurorepair responses after cerebral ischemia in mice (Rosell et al., 2013). Our present findings suggest that EPCs might exert paracrine effects on cerebral ischemic injury in addition to their direct angiogenic effects, and chronic exposure to subtherapeutic chlortetracycline reduced the therapeutic effect of EPC-conditioned media on cerebral ischemic injury reduction in mice.

## 4 Discussion

This study showed the first evidence that chronic subtherapeutic antibiotic exposure impaired EPC-mediated ischemic angiogenesis and aggravated cerebral ischemic injury in mice.

EPCs have been demonstrated to participate in both vasculogenesis and vascular homestasis, and have been used to successfully restore endothelial function and enhance angiogenesis in ischemic brain tissue (Zhao et al., 2013; Fan et al., 2010; Marrotte et al., 2010). In the present study, it was found, for the first time, that chronic treatment with chlortetracycline, penicillin or vancomycin could impair EPC functions, reduce the angiogenesis in ischemic brain, and aggravate long-term stroke outcome in mice. Furthermore, we found that transplantated EPCs from chronic antibiotic-treated mice showed a lower therapeutic effect on cerebral ischemic injury reduction and local angiogenesis promotion compared to those from control mice, and the EPCs from the donor animals could integrate into the recipient ischemic brain. In addition, it has been showed that EPC-conditioned media, which contain growth factors (including PDGF, VEGF, HGF, etc), can accelerate diabetic wound healing, and an intravenous injection of EPC-conditioned media can promote angiogenesis and improve neurological outcome after cerebral ischemia in mice (Marrotte et al., 2010; Rosell et al., 2013). Consistent with these results, our present findings suggested that, in additional to their direct angiogenic effects, EPCs might exert paracrine effects on cerebral ischemic injury reduction, which could be impaired by chronic antibiotic exposure. Therefore, the impairment of both EPC-mediated ischemic brain angiogenesis and EPCs' paracrine effects, as observed in the present study, may partly contribute to the aggravated cerebral ischemic injury produced by chronic antibiotic exposure in mice. Other possible mechanisms of this response remain to be investigated in further studies.

Benakis C et al. demonstrated that treatment with therapeutic dose of antibiotics amoxicillin and clavulanic acid (AC) (1000 mg/l in drinking water) for 2 weeks reduced the gut microbiota diversity in AC-sensitive mice (Benakis et al., 2016). However, low-dose penicillin (6.67 mg/l in drinking water), delivered from birth, was found to increase gut microbiota phylogenetic diversity at 4 weeks in mice (Cox et al., 2014). Thus, it is implied that different dosage and timing of antibiotic treatment could exert different effects on gut microbiota diversity. These findings might give a potential hint to the contradictory effects of antibiotic exposures on ischemic brain injury found between the present work and Benakis C and colleagues' study, which remains to be test by further studies.

It has been shown that eNOS and MnSOD critically regulate EPC function (Marrotte et al., 2010; Xie et al., 2010). In addition, TSP-2 can inhibit angiogenesis, and has been proposed as an inhibitor of endothelial cell and EPC function (Bae et al., 2013). Thus, the decreased eNOS and MnSOD levels together with the increase of TSP-2 levels, as observed in the present study, might partly contribute to the EPC dysfunction in antibiotics-treated mice.

In addition, since EPCs has been implicated in playing an important role in vascular repair and revascularization in various ischemic organs besides brain tissue, and EPC is inversely correlated with a number of cardiovascular risk factors (Zhao et al., 2013; Fan et al., 2010; Marrotte et al., 2010), EPC dysfunction produced by chronic antibiotic exposure might also aggravate other ischemic diseases except stroke, such as acute myocardial infarction, peripheral vascular ischemic diseases, and so on, which remains to be test by further studies.

In summary, chronic exposure to subtherapeutic chlortetracycline, penicillin or vancomycin could aggravate cerebral ischemic injury in mice, which might be partly attributed to the impairment of both EPC-mediated ischemic brain angiogenesis and EPCs' paracrine effects. These findings reveal a previously unrecognized impact of chronic subtherapeutic antibiotic exposure on ischemic injury, which warrants further attention in daily life.

## Funding Sources

This work was supported by the National Key Basic Research Program of China (973 Program; 2014CB542403), the National Natural Science Foundation of China (81370253, 81170115 and 81603097), and The Qihang Project Foundation of Shanghai East Hospital, Tongji University (DFQH-Q4).

## Conflicts of Interest

The authors declare no competing financial interests.

## Author Contributions

H.H.X. conceived and designed the experiments. X.H.D., C.P., Y.Y.Z., Y.L.T., X.T. and C.Z. performed the experiments. H.H.X., X.H.D. and A.F.C. analyzed the data. H.H.X. wrote the manuscript.
